# The Role of microRNA-19b, microRNA-21, and microRNA-208a in diagnosis of heart failure

**DOI:** 10.1186/s12872-025-05272-9

**Published:** 2025-12-01

**Authors:** Waleed M. Fathy, Belal A. Montaser, Waleed A. Ibrahim, Doaa M. Salah, Noran T. Aboelkhair

**Affiliations:** 1 Clinical Pathology Department, Faculty of Medicine, Menoufia University, Shebin El-Kom, Egypt; 2 Cardiology Department, Faculty of Medicine, Menoufia University, Shebin El-Kom, Egypt

**Keywords:** Heart failure, miR, 19b, miR, 208a, miR, 21, NT, ProBNP

## Abstract

**Background:**

Heart failure (HF) is a fast-growing public health concern that is linked to substantial morbidity and mortality as well as a poor quality of life. There is no specific biomarker that can be used to detect early HF with preserved ejection fraction (EF). MicroRNAs (miRs) are known to be involved in the development of HF.

**Aim:**

To investigate the plasma level of miR-19b, miR-21, and miR-208a and their relevance in early diagnosis of HF.

**Subjects and methods:**

In this study, 120 participants were divided into four groups: 30 controls in Group I, 30 HF patients with preserved EF in Group II, 30 HF patients with mildly reduced EF (HFmrEF) in Group III, and 30 HF patients with reduced EF (HFrEF) in Group IV. Real-time PCR was used to examine the expression levels of miR-19b, miR-21, and miR-208a, and ELISA was used to assess the NT-proBNP level.

**Results:**

Compared to controls, HF patients' groups exhibited a substantial sequential rise in miR-21 and miR-208a expression (*p* < 0.001). However, HF patients had considerably lower levels of miR-19b expression compared to controls (*p* < 0.001). The cutoff value of miR-19b to discriminate HFmrEF from HFpEF was 11.2 (93.3% sensitivity, 86.7% specificity) (*p* < 0.001). In contrast, miR-21 had a cutoff value of 5.5 (70% sensitivity and 76.7% specificity), and miR-208a had a cutoff point of > 10.3 (93.3% sensitivity and 93.3% specificity).

**Conclusion:**

MiR-19b, miR-208a and miR-21 could be useful biomarkers with high sensitivity and specificity for early detection of HF and in discrimination between HFmrEF and HFpEF.

**Supplementary Information:**

The online version contains supplementary material available at 10.1186/s12872-025-05272-9.

## Introduction

Heart failure (HF) is a serious clinical condition with elevated rates of morbidity, death, and medical costs. The heart's reduced capacity to pump blood effectively results in a group of symptoms known as HF [1]. Myocardial injury results in HF for a variety of reasons, such as diabetes, hypertension, and ischemic heart disease. Moreover, some of the less common causes of HF include toxins, cardiomyopathies, myocarditis, valvular disorders, and cardiotoxic drugs. Patients with HF show symptoms such as dyspnea due to pulmonary congestion, as well as peripheral oedema and ascites due to impaired drainage. Weakness, nausea, and appetite loss are common manifestations [2]. HF is caused by alterations to the heart's function, structure, or both. The diagnosis is based on symptoms, physical findings, echocardiography, and circulating natriuretic peptides [3]. The three types of HF are HF with reduced EF (HFrEF), HF with moderately reduced EF (HFmrEF), and HF with preserved EF (HFpEF; each has to be differentiated from others using state-of-the-art echocardiography examination. Serum N-terminal pro-brain natriuretic peptide (NTproBNP) is primarily listed as a biomarker for HF in the guidelines and plays a crutial role in HF diagnoses, but novel specific biomarkers are needed for the early diagnosis of various HF subtypes [4].

MicroRNAs (MiRs) are small, single-stranded, non-coding RNAs of 22–24 nucleotides in length. They affect target gene expression on the post-transcriptional level which is important to maintain physiological functions of the cells and tissues, but it may lead to the development of various pathologies. Several studies have indicated that numerous miRNAs have been implicated in the progress of cardiovascular disorders [5]. MiR-19b has been found as an integral component of the miR-17–92 gene cluster and modulates various target genes in human tissue. It was reported that miR-19b can safeguard cardiomyocytes from apoptosis, so miR-19b has been linked to different cardiovascular disorders [6].

MiR-21 has been identified to be a diagnostic marker for several cardiac conditions, such as diabetic cardiomyopathy, coronary artery disease, myocardial ischemia reperfusion injury, and myocardial infarction. MiR-21 is produced in response to cardiac stresses, mediating stress-correlated signaling pathways and preventing cardiomyocytes from undergoing apoptosis [7]. MiRNA expression profiling studies validated numerous significant miRNAs, including miR-21. These miRNAs could be employed as non-invasive biomarkers and HF medication targets [8]. The expression of miR-21 augmented with the severity of an HF exacerbation and was shown to be highly expressed in cardiac fibroblasts rather than myocardiocytes. It has been reported that this miRNA molecule prevents cardiac fibroblasts from dying and increases the mitogen-activated protein kinase signaling pathway's signal transduction, resulting in myocardial fibrosis and cardiac hypertrophy—two significant pathological factors linked to the pathogensis of HF [9].

MiR-208 plays a key role in cardiac cell stress, electrical stimulation, and hormone release. MiR-208 increases cell damage and promotes cell death, which has been related to ischemic heart disease, cardiac fibrosis, and hypertrophy [10]. It has been documented that a variety of myocardial disorders are linked to considerable changes in the plasma level of heart-associated miRNA-208b, which is progressively higher during acute HF, acute viral myocarditis, and acute myocardial infarction [11]. It is important to note that while the intracellular roles of microRNAs such as miR-19b, miR-21, and miR-208 involve regulation of apoptosis, fibrosis, and signaling pathways within cardiac cells, in the context of our study, these microRNAs are assessed as extracellular biomarkers in plasma, serving as indicators rather than effectors of cardiac pathology. Thus, the aim of this study was to assess the contribution of miR-19b, miR-21, and miR-208a to the early detection of HF.

## Subjects and methods

### Subjects

This case–control study was conducted on 120 subjects (80 males and 40 females). Group I comprised 30 seemingly healthy subjects who were matched for gender and age with the other groups; group II included 30 HF patients with preserved EF (HFpEF) ≥ 50%; group III included 30 HF patients with EF 41–49% and a modestly decreased EF (HFmrEF); and group IV included thirty HF patients with EF ≤ 40% and a reduced EF (HFrEF). Left ventricular EF was measured using the biplane Simpson’s method on two-dimensional echocardiography (GE Vivid 9 Ultrasound Machine, Chicago, USA), and blood sampling was done after diagnosis for classifications of patients. Medical history was taken from all participants and they underwent general and local examination. Each participant, provided informed consent and the study was approved by the Regional Ethical Committee (IRP: 9/2021CPATH21).

Patients with malignancies, autoimmune disorders, persistent infections, and chronic inflammatory conditions (as rheumatoid arthritis, chronic obstructive pulmonary disease, inflammatory bowel disease, and chronic viral infections such as hepatitis B or C) were excluded from the study. Patients with atherosclerosis were not excluded unless they had additional evidence of systemic inflammatory disease.

### Methods

#### Collection and sample preparation

Under sterile conditions, seven milliliters of blood were drawn and split as follows: Two milliliters of blood were added to an EDTA tube for Complete blood count (CBC) analysis on a Sysmex XN-1000 Automated Hematology Analyzer (Sysmex, Japan), and for HbA1c on Capillary Octa 3 capillary electrophoresis (Sebia, London). Three milliliters were added to a plain tube, and serum was separated for assessment of urea, creatinine and liver functions on a Beckmann Chemistry-autoanalyzer (AU 680 Beckmann, USA). Two milliliters of blood were placed in an EDTA container, the blood samples were centrifuged for 15 min at 1,500 × g using a refrigerated centrifuge at 4 °C to ensure optimal preservation of RNA integrity. The plasma was carefully collected, aliquoted and immediately stored at − 80 °C; one for NT-proBNP assay by ELISA according to producer directions (Sunlong, China), and the other for miRNA analysis.

#### miRNA analysis

The expressions of miR-19b, miR-21 and miR-208a were analyzed using quantitative real-time PCR (qRT-PCR). The RNA extraction was performed in accordance with the instructions on the miRNeasy-extraction kit (Qiagen, USA). A NanoDrop 2000 spectrophotometer (Thermo Scientific, USA) was used to assess the quantity of RNA at 260/280 nm. 2 μl of 5X miRCURY SYBR Green-RT Reaction Buffer, 4.5 μl of RNase-free water, 1 μl of 10 × miRCURY-RT Enzyme Mix, 0.5 μl UniSp6 RNA spike-in, and 2 μl of Template RNA were used to perform reverse transcription in a 10 μl reaction volume. After 60 min of incubation at 42 °C and five minutes at 95 °C, the mixture was kept at 4 °C until it was used.

Mercury LNA (hsa-mir-19b-3p, Qiagen, USA), Mercury LNA (hsa-mir-21-5p, Qiagen, USA), Mercury LNA (hsa-mir-208a-3p, Qiagen, USA), and the housekeeping gene (RNU6B, Qiagen, USA) were used as the control. The expression levels of the plasma miR-19b, miR-21, and miR-208a were assessed by qRT-PCR using particular MiScript Primers. A final volume of 10 μl was created by combining three microliters of cDNA template with five microliters of SYBR Green Master Mix (Qiagen, USA), 0.05 µl of ROX Reference Dye, one microliter of each of the primers for miR-19b, miR-21, miR-208a, and RNU6B for qRT-PCR, and 1µl of nuclease-free H_2_O.

A 7500 Real-Time PCR system (Applied Biosystems, USA) was used for all reactions, which were run for two minutes at 95 °C, followed by 40 cycles at 95 °C for 10 s and 56 °C for 60s. The cycle threshold (CT) in real-time PCR is the number of cycles needed for the fluorescent signal to reach the threshold. The ΔCt value, which was obtained by deducting the RNU6B CT values from the CT values of the microRNAs under investigation, was used to compute the expression of miR-19b, miR-21, and miR-208a. Because of the inverse relationship between ΔCt and miRNA expression level, higher ΔCt values were associated with lower miRNA levels. Using the 2^–ΔΔCt^ approach developed by Livak and Schmittgen, where ΔΔCt = ∆Ct (sample) − ∆Ct (control average), the relative quantitative levels of particular miRNAs were ascertained.

#### Statistical analysis

IBM SPSS version 20.0 was used to conduct statistical analysis. Descriptive statistics, such as mean and standard deviation, were used for parametric variables. Using chi-squared analysis on numerical data The one-way ANOVA test is used to assess the correlation between more than two groups for normally distributed quantitative variables, the Mann–Whitney test is used to compare more than two groups, the Kruskal Wallis test is used to compare more than two studied groups, and the Post Hoc test (Bonferroni test**)** is used for pairwise comparisons. The Spearman coefficient was used to ascertain the association between two quantitative variables that have an irregular distribution. The receiver operating curve (ROC) was constructed, and the area under the curve (AUC) was computed, to determine the sensitivity and specificity. The P-value was considered statistically significant if it was 0.05 or less.

## Results

### Laboratory results and echocardiography

Groups IV and III had significantly lower EF than group II (*P* < 0.001) (Table 1), and levels of total cholesterol, LDL-C, triglycerides ALT, AST, urea, and HbA1c were significantly higher in HF groups than in controls (*P* < 0.001), but lower in HDL-C and hemoglobin (Hb) (*P* < 0.001). Furthermore, pro-BNP levels showed statistically significant difference, with group IV having significantly higher levels than group III, and group III having significantly higher levels than both group II and the controls (*P* < 0.001) (Table 2).

**Table 1:** Demographic and clinical data of the studied groups

**Studied variables**	**Group I** **(Controls)** ***N* = 30**	Group II (HFPEF) *N* = 30	Group III (HFmrEF) *N* = 30	Group IV (HFREF) *N* = 30	Test of sig	*P* value
Age/years Mean ± SD	50.7 ± 0.79	50.6 ± 0.72	51.06 ± 0.96	50.8 ± 0.77	F 0.68	= 0.563
Gender Male Female	**No (%)** 18(60.0) 12(40.0)	**No (%)** 20(66.7) 10(33.3)	**No (%)** 22(73.3) 8(26.7)	**No (%)** 20(66.7) 10(33.3)	χ^2^ 0.600	= 0.896
Hypertension Yes No	0(0.00) 30(100)	18(60.0) 12(40.0)	18(60.0) 12(40.0)	16(53.3) 14(46.7)	χ^2^ 15.4	> 0.001*
Diabetes mellitus Yes No	0(0.00) 30(100)	16(53.3) 14(46.7)	16(53.3) 14(46.7)	16(53.3) 14(46.7)	χ^2^ 13.3	= 0.004*
Ejection fraction (%) Mean ± SD	68.1 ± 2.68	62.0 ± 1.81	53.1 ± 1.76	36.7 ± 5.71	F 240.0	** > 0.001**
Post hoc (Bonferroni test)	P1:0.001* P2: 0.001* P3:0.001* P4:0.001* P5:0.001* P6:0.001*					

*No* number,% percentage, *SD* Standard deviation, *F* ANOVA test χ^2^: Chi square test, *: Statistically significant

Group I: Controls

Group II: Heart failure patients with preserved ejection fraction (HFpEF)

Group III: Heart failure patients with mild reduced ejection fraction (HFmrEF)

Group IV: Heart failure patients with reduced ejection fraction (HFrEF(

P1: Comparison between group I & group II

P2: Comparison between group I & group III

P3: Comparison between group I & group IV

P4: Comparison between group II & group III

P5: Comparison between group II & group IV

P6: Comparison between group III & group IV

**Table 2:** Laboratory data of the studied groups

**Studied variables**	**Group I** **(Controls)** ***N* = 15**	**Group II** **(HFPEF)** ***N* = 15**	**Group III** (**HFmrEF)** ***N* = 15**	**Group IV** **(HFREF)** ***N* = 15**	**Test of sig**	***P* value**
**Total cholesterol** Mean ± SD	181.0 ± 6.97	224.4 ± 22.3	218.6 ± 21.0	233.9 ± 17.6	F = 51.4	< 0.001*
**Post hoc (Bonferroni test)**	P1:0.001* P2: 0.001* P3:0.001* P4:0.5 P5: 0.17 P6:0.006					
**LDL-C** Mean ± SD	93.3 ± 5.08	151.8 ± 19.0	146.1 ± 20.1	162.1 ± 16.7	F = 108.6	< 0.001*
**Post hoc (Bonferroni test)**	P1: 0.001 P2: < 0.001* P3:0.001* P4: 0.5 P5:0.07 P6:** 0.001**					
**Triglycerides** Mean ± SD	130.8 ± 10.4	155.9 ± 23.8	151.8 ± 19.0	156.8 ± 23.0	F = 11.7	< 0.001*
**Post hoc (Bonferroni test)**	P1: 0.001* P2: 0.001* P3: 0.001* P4:0.8 P5:0.9 P6:0.7					
**HDL–C** Mean ± SD	61.3 ± 4.89	41.5 ± 5.01	42.0 ± 5.01	40.6 ± 4.32	F = 136	< 0.001*
**Post hoc (Bonferroni test)**	P1:0.001* P2: 0.001* P3:0.001* P4:0.9 P5:0.8 P6:0.6					
**ALT** Mean ± SD	26.6 ± 2.97	36.6 ± 5.64	36.1 ± 7.80	39.8 ± 8.39	F = 10.7	< 0.001*
**Post hoc (Bonferroni test)**	P1:0.001* P2: 0.002* P3:0.001* P4:1.00 P5:1.00 P6:0.834					
**AST** Mean ± SD	30.6 ± 4.06	40.2 ± 5.27	40.2 ± 7.61	44.1 ± 7.96	F = 11.9	< 0.001*
**Post hoc (Bonferroni test)**	P1:0.001* P2: 0.001* P3:0.001* P4:1.00 P5:0.669 P6:0.669					
**Urea** Mean ± SD	28.6 ± 3.88	37.6 ± 5.77	35.2 ± 5.68	42.6 ± 9.12	F = 12.3	< 0.001*
**Post hoc (Bonferroni test)**	P1:0.002* P2: 0.037* P3: 0.001* P4:1.00 P5:0.222 P5:0.016*					
**Creatinine** Mean ± SD	0.91 ± 0.15	1.20 ± 0.29	1.17 ± 0.24	1.31 ± 0.41	F = 4.80	0.005*
**Post hoc (Bonferroni test)**	P1:0.061 **P2: 0.116** P3: 0.003* P4:1.00 P5:1.00 P6:1.00					
**HbA1c** Mean ± SD	5.12 ± 0.38	7.00 ± 1.32	6.87 ± 1.29	6.91 ± 1.30	F = 9.35	< 0.001*
**Post hoc (Bonferroni test)**	P1:0.001* P2: 0.001* P3:0.001* P4:1.00 P5:1.00 P6:1.00					
**Hb** Mean ± SD	14.5 ± 1.24	12.2 ± 1.40	11.9 ± 1.26	11.5 ± 1.36	F = 15.1	< 0.001*
**Post hoc (Bonferroni test)**	P1:0.001* P2: 0.001* P3:0.001* P4:1.00 P5:1.00 P6:1.00					
**WBC** Mean ± SD	6.86 ± 1.38	7.00 ± 1.40	7.94 ± 1.82	6.36 ± 1.62	F = 2.64	0.058
**Platelet** Mean ± SD	251.8 ± 58.5	305.9 ± 84.6	261.2 ± 89.4	284.8 ± 88.9	F = 1.34	0.270
**NT-proBNP** (pg/ml) Mean ± SD	85.3 ± 9.8	643 ± 248	801 ± 89.8	913 ± 124.6	F 196.6	< 0.001*
**Post hoc (Bonferroni test)**	P1:0.001* P2: 0.001* P3:0.001* P4: 0.001* P5: 0.001* P6: 0.001*					

*SD* Standard deviation, F: ANOVA test, *: Statistically significant

Group I: Controls

Group II: Heart failure patients with preserved ejection fraction (HFpEF)

Group III: Heart failure patients with mild reduced ejection fraction (HFmrEF)

Group IV: Heart failure patients with reduced ejection fraction (HFrEF(

P1: Comparison between group I & group II

P2: Comparison between group I & group III

P3: Comparison between group I & group IV

P4: Comparison between group II & group III

P5: Comparison between group II & group IV

P6: Comparison between group III & group IV

### miRNA plasma levels

**Fig. 1:**
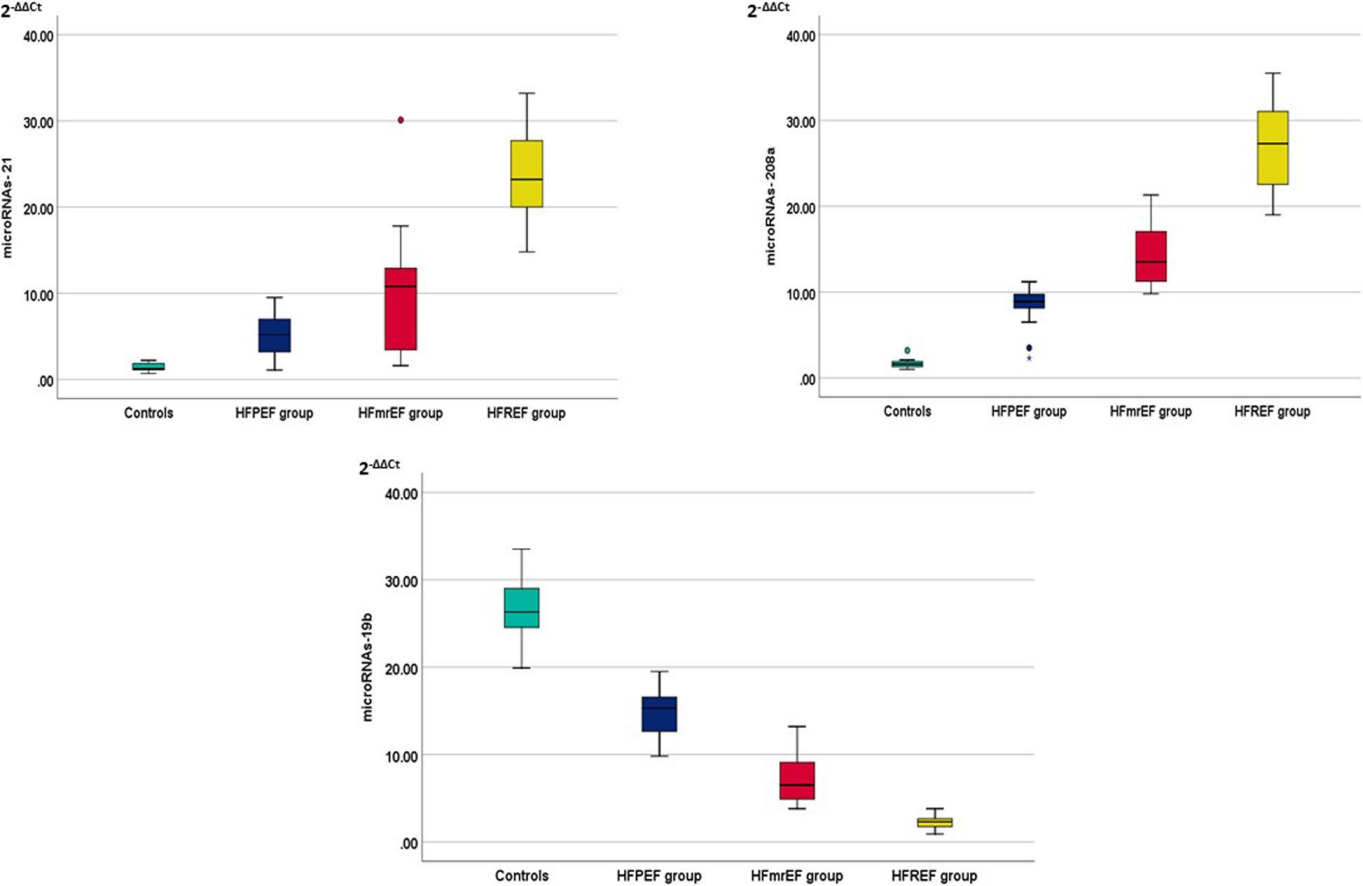
Plot graph for miR-19b, miR-21 and miR-208a expression (2^−ΔΔCt^) among the studied groups

**Fig. 2:**
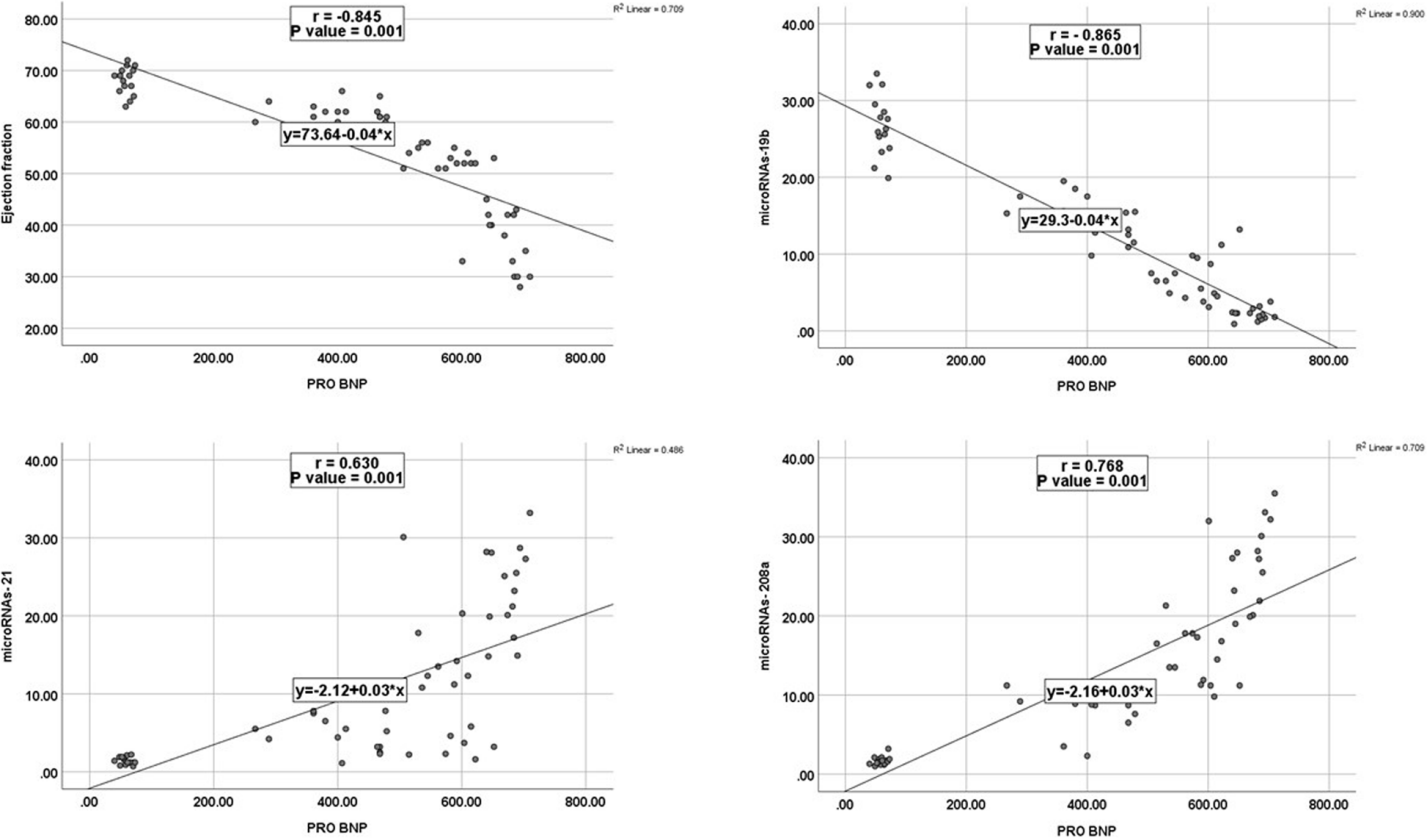
Correlation between pro-BNP level and EF, miR-19, miR-21, miR-208a

**Fig. 3:**
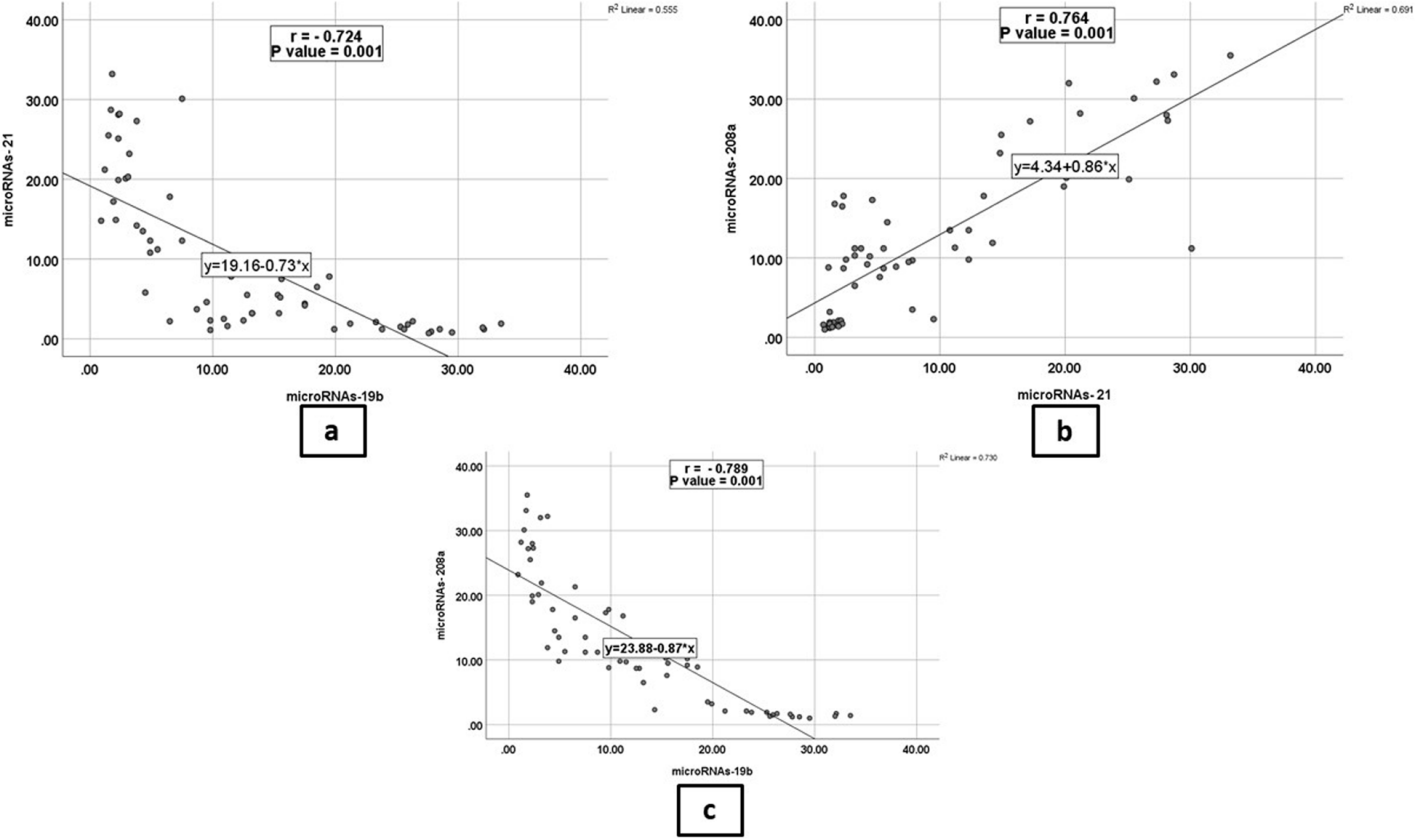
**a** Correlation between miR-19b and miR-21. **b** Correlation between miR-21 and miR-208a. **c** Correlation between miR-208a and miR-19b

MiR-21 and miR-208a plasma levels were significantly more elevated in HF groups compared to controls. Additionally, group IV had higher levels than group III, while group III had higher levels than group II (*P* < 0.001). All HF groups showed decreased plasma levels of miR-19b compared to controls; they were lower in group IV than in group III, and lower in group III than in group II (*P* < 0.001) **(**Fig. 1). MiR-21 and 208a showed a significant positive correlation with the NT-proBNP level. Meanwhile, EF and miR-19b exhibited a significant negative correlation with NT-proBNP level (*p* < 0.001) (Fig. 2). There was a significant positive correlation between EF and miR-19b expression, and there were significant negative correlations between EF and both miR-21 and miR208a (*p* < 0.001). Additionally, there was a significant positive correlation between miR21 and miR-208a expression (*p* < 0.001), meanwhile there were significant negative correlations between the level of miR-19b and both miR21 and miR-208a (*p* < 0.001) (Fig. 3), (Supp Tables 1, 2, 3).

**Table 3:** Validity for NT-proBNP miR-19b, miR-21, and miR-208a to discriminate heart failure patients

**Studied variables**	**AUC**	***P*** value	**95%CI**		**Cutoff point**	**Sensitivity (%)**	**Specificity (%)**	**PPV** **(%)**	**NPV** **(%)**
			**Lower bound**	**Upper bound**					
NT-proBNP (pg/ml)	0.970	< 0.001*	0.931	1.00	> 97	95.5%	86.7%	97%	100%
miR-19b (2^−ΔΔCt^)	1.00	< 0.001*	1.00	1.00	< 19.9	100%	100%	100%	100%
miR-21 (2^−ΔΔCt^)	0.974	< 0.001*	0.935	1.00	> 2.15	95%	93%	97%	87%
miR-208a (2^−ΔΔCt^)	0.999	< 0.001*	0.994	1.00	> 2.75	97%	93%	97%	93%
NT-proBNP + miR-19b + miR-21 + miR—208a	-	**-**	-	-	-	100%	100%	100%	100%

*AUC* Area under the curve, *CI* Confidence interval, *PPV* Positive predictive value, *NPV* Negative predictive value, *: Statistically significant

### AUC analysis

**Fig. 4:**
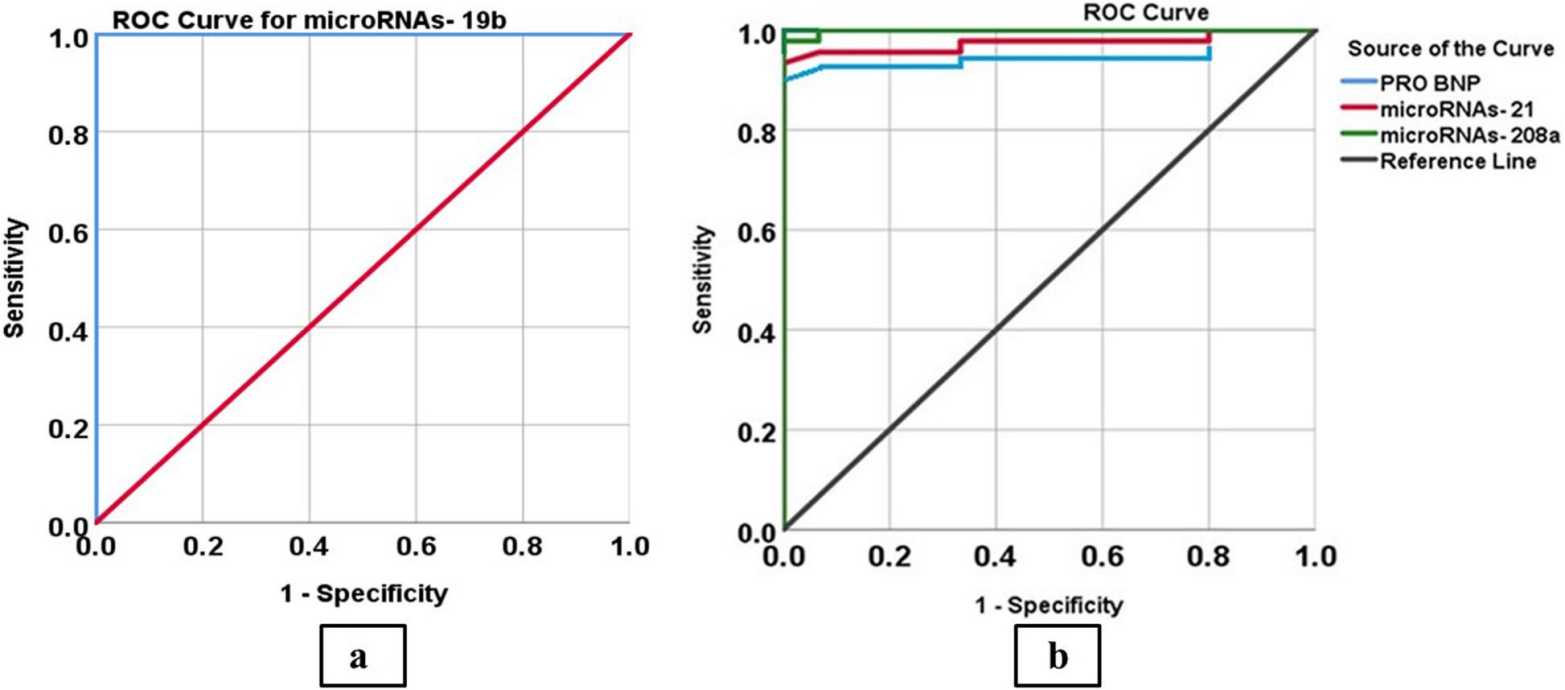
**a** Roc curve for sensitivity and specificity of miR-19b in detection of HF. **b** ROC curve for sensitivity and specificity of NT-proBNP, miR-21, and miR-208a in detection of HF

NT-proBNP had a cutoff value of 97 pg/ml, 95.5% sensitivity, 86.7% specificity, 0.970 AUC and 95% confidence interval (CI) was 0.931–1.00 for the diagnosis of HF. MiR-21 cutoff point of 2.15 displayed 0.974 AUC, CI was 0.935–1.00, 95% sensitivity, and 93% specificity. Also, miR-208a demonstrated 0.999 AUC, CI was 0.994–1.00, 97% sensitivity, and 93% specificity at a cutoff point 2.75. MiR-19b with cutoff 19.9 had 100% sensitivity, 100% specificity, 1 AUC and CI was 1.0–1.0. The combination of NT-proBNP and all studied miRNAs yielded 100% sensitivity and specificity in distinguishing patients with HF from controls (Fig. 4), (Tables 3, 4). In addition, miR-19b and miR-208a had higher sensitivity and specificity than NT-proBNP to discriminate HFmrEF from HFpEF. While miR-19b, miR-21 and miR-208a had higher sensitivity and specificity than NT-proBNP to discriminate HFmrEF from HFrEF (Supp Tables 4, 5).

**Table 4:** DeLong’s test to statistically compare AUCs of different biomarker

Studied variables	AUC	*P* value
miR-19b vs NT-proBNP	1.00–0.970	0.342
miR-19b vs miR-21	1.00—0.974	0.244
miR-19b vs miR-208a	1.00 −0.999	0.921

## Discussion

The prevalence of HF has increased during the last few decades, with major social, economic, and health consequences. Target gene expression is influenced by miRNAs, which makes them potential biomarkers for a variety of cardiovascular conditions [12].

MiR-19b, miR-21 and miR-208a have been shown to play a role in the HF pathophysiology [13–15]. Novel biomarkers are needed in HF diagnosis, so the current study investigated the relationship between the expression of circulating miR-19b, 21, 208a, and HF since finding sensitive and specific biomarkers for HF is an essential area of cardiovascular research.

In the current study, NT-proBNP was significantly higher in HFrEF patients compared to HFmrEF, HFpEF and controls. These findings were consistent with those of Podzolkov et al. [16], Tromp et al. [17] and Yu et al. [18], who discovered that the HFrEF group had considerably more NT-proBNP than the controls. Natriuretic peptides are hormones that are produced in the heart and have diuretic, natriuretic, and vasodilatory functions. In response to elevated heart wall stress, they are released into the bloodstream. They have strong diagnostic potential for distinguishing between cardiac and non-cardiac dyspnea, as well as predictive relevance in terms of death and repeated hospitalizations in HF patients. In individuals without previous cardiovascular illness, elevated NT-proBNP levels are a strong predictor of eventual heart failure (Melendo-Viu et al., [19]; McKechnie et al., [20])

In this study, miR-21 was significantly up-regulated in HFrEF patients compared to HFmrEF, and in HFmrEF compared to HFpEF, with all HF groups showing significantly higher expression than controls. MiR-21 at a cutoff point 2.15 had 95% sensitivity 95% and 93% specificity to diagnose HF. Likewise, Shen et al., [8], Kuai et al., [21] found that miR-21 was significantly up-regulated in HF patients than controls. In addition, Zhang et al., [22] found that microR-21 had a sensitivity of 100%, specificity of 97.5%, and AUC 0.948. Also, Ding et al., [23] found that miR-21 expression had a sensitivity of 89.7%, specificity of 82.8%, and AUC of 0.944 for the identification of patients with HF. The pathophysiological processes that control the onset and development of HFpEF include myocardial cell fibrosis and pathological hypertrophy, which raise left ventricular filling pressures and ultimately result in the HF syndrome. Since miR-21 is linked to myocardial fibrosis, there have been numerous studies looking into ways to block miR-21 in order to stop fibrosis [24]. The consistency across studies may reflect the vital role of miR-21 in mediating cardiac fibrosis and hypertrophy, both of which are key features of HF pathophysiology. Furthermore, the dual role of miR-21 —promoting fibrosis via fibroblast activation while protecting cardiomyocytes from apoptosis —may explain its persistent elevation across all HF phenotypes [24]. However, our study is among the first to demonstrate a gradient of miR-21 expression across HF subtypes (HFrEF > HFmrEF > HFpEF), suggesting that miR-21 levels may reflect disease severity or degree of structural remodeling. Its strong performance as a plasma biomarker reinforces its potential not only in diagnosis but also in stratifying HF phenotypes, which has important clinical implications given the differing treatment responses and outcomes among HF subtypes.

The current study found that miR-208a plasma levels were significantly elevated in HF groups compared to controls and that they were higher in HFrEF patients compared to HFmrEF patients and HFmrEF patients compared to HFpEF patients. MiR-208a at a cutoff point 2.75 had 97% sensitivity and 93% specificity. In accordance to these results, Li et al., [25] found that miR-208a was significantly up-regulated in HFrEF patients than in HFpEF patients. Also, Kuai et al., [21] found that miR-208a was significantly up-regulated in HF patients than controls. However, Li et al., [25] found that miR-208a had a 68% sensitivity, 90.2% specificity, and 0.83 AUC. MicroRNA-208a's function is linked to the synthesis of heavy myosin chains and heart development. In dilated cardiomyopathy patients, microRNA-208a up-regulation was positively correlated with left ventricular end-systolic volume index but negatively correlated with EF [26]. The strong diagnostic performance of miR-208 can be attributed to its cardiac specificity—it is encoded by the MYH6 and MYH7 genes and released in response to cardiomyocyte injury, electrical stimulation, and hypertrophic stress. Its elevation in plasma likely reflects direct myocardial cell damage or remodeling processes, which are more pronounced in HFrEF and HFmrEF compared to HFpEF [10]. Our study stands out for showing a correlation between plasma miR-208 levels and the HF subtype, which may indicate a relationship between circulating levels and the degree of underlying cardiac stress. This strengthens the body of literature by indicating that miR-208 may be useful for differentiating between phenotypes with varying degrees of systolic dysfunction in addition to detecting a presence of HF.

Our study findings showed that miR-19b expression was significantly lower in HF groups compared to controls, with HFrEF patients exhibiting significantly lower levels than HFmrEF patients and HFmrEF patients exhibiting lower levels than HFpEF patients. MiR-19b at a cutoff < 19.9 had 100% sensitivity and 100% specificity to diagnose HF. In accordance with these results, Zhang et al., [27], Shen et al., [8] found that miR-19b was significantly decreased in HFrEF patients than in HFPEF patients., and Zhang et al., [27] found that the miR-19b had a sensitivity of 77% and a specificity of 79%. Some research reported reduced expression of miR-19b in advanced HF, particularly in HFrEF, likely due to extensive cardiomyocyte loss, maladaptive remodeling, and exhaustion of compensatory signaling mechanisms. miR-19b is part of the miR-17–92 cluster, which is involved in regulating apoptosis, inflammation, and survival signaling in cardiomyocytes [28]. In early or less severe stages of cardiac stress, such as HFpEF, miR-19b may be upregulated to promote cell survival and suppress apoptosis. However, in late-stage or severe heart failure, like HFrEF, chronic stress, inflammation, and cellular exhaustion may lead to downregulation of miR-19b, possibly reflecting loss of cardioprotective signaling capacity. This shift may also correspond with fibrotic remodeling and loss of viable myocardium, where the decreased presence of cardiomyocytes leads to lower intracellular production and release of miR-19b into plasma [29].

The results demonstrated a significant positive relationship between NT-proBNP levels and miR-21 and 208a. Additionally, there was a substantial negative correlation between the NT-proBNP level and EF and miR-19b. Likewise, Zhang et al. [27] found a strong inverse relationship between NT-proBNP and miR-19b. Additionally, Li et al. [25] stated a significant positive association between NT-proBNP and miR-208a. Furthermore, Landolfo et al., [30] reported that the NT-proBNP level and EF had a significant opposing correlation. Additionally, the data showed a significant negative correlation between the levels of NT-proBNP and EF and miR-208a. Likewise, Li et al. [25] stated a significant adverse relationship between the pro-BNP level and the miR-208a level. This study's use of miR-19b produced the best results for HF diagnosis (with higher sensitivity and specificity) and HFpEF/HErEF differentiation. Given that fibrosis development is more significantly associated with progressive HFrEF than HFpEF, it can be speculated as a possible explanation of our results.

In this study, miR-19b and miR-208a had higher sensitivity and specificity than NT-proBNP to discriminate HFmrEF from HFpEF. While miR-19b, miR-21 and miR-208a had higher sensitivity and specificity than NT-proBNP to discriminate HFmrEF from HFrEF. The integration of the three miRNAs with NT-proBNP significantly improved diagnostic accuracy, achieving 100% sensitivity and specificity. These findings provide validity to the potential advantage of multi-marker approaches in medical practices, aligning with recent efforts to improve biomarker-based diagnosis in HF.

MiRNAs have displaced conventional protein-based diagnostics as extremely promising indicators for CVD. In contrary to proteins, which exhibit variable expression levels and are susceptible to secondary influences, miRNAs exhibit remarkable stability in body fluids such as saliva, plasma, and blood. They are optimal candidates for the precise and prompt detection of CVD because of their tiny size and resistance to deterioration [31].

Moreover, miRNAs directly regulate cardiovascular disease, including fibrosis, apoptosis, and inflammation, providing mechanistic information that protein biomarkers usually do not. The sensitivity of certain miRNAs to various cardiovascular diseases allows for accurate risk assessment, disease classification, and therapeutic response tracking. Additionally, improvements in quantitative PCR and high-throughput sequencing technologies have made it possible to discover miRNAs quickly and affordably, which has increased their therapeutic relevance. Therefore, miRNAs have a great potential to improve on current biomarkers, resulting in more precise and customized methods of diagnosing and managing CVD [32].

This study adds to current knowledge by showing that circulating miR-21, miR-208 and miR-19b levels correlate with HF phenotype, making it promising candidates for both diagnosis and subtype differentiation. This may offer additional clinical value in the personalized management of HF. The limitations of this study comprise its relatively small sample size and its single-centered ethnicity design. Although both male and female participants were included, the sample size did not allow for a detailed analysis of gender-specific differences in biomarker expression. Consequently, further larger-scale investigation is recommended to confirm our findings and to explore potential sex-related variations in diagnostic performance.

## Conclusion

Our findings contribute new evidence supporting the role of circulating miR-21, miR-208 and miR-19b as sensitive non-invasive cardiac biomarkers and possible indicators of HF progression severity if validated on the larger cohorts. MiR-19b and miR-208a had higher sensitivity and specificity than NT-proBNP to discriminate HFmrEF from HFpEF, while miR-19b, miR-21 and miR-208a had higher sensitivity and specificity than NT-proBNP to discriminate HFmrEF from HFrEF. Further research on larger cohort is needed to validate the results and draw clear conclusions.

## Supplementary Information

Supplementary Material 1

## Data Availability

The data sets generated and analyzed during the current study are available from the corresponding author upon reasonable request.

## Abbreviations

AUC: Area under the curve
CT: Cycle threshold
EF: Ejection fraction
HF: Heart failure
HFmrEF: Moderately reduced ejection fraction
HFpEF: Preserved ejection fraction
HFrEF: Reduced ejection fraction
MiRs: MicroRNAs
qRT-PCR: Quantitative real-time PCR
ROC: Receiver operating curve
