# Supplementary material for: The Role of microRNA-19b, microRNA-21, and microRNA-208a in diagnosis of heart failure
Source: BMC Cardiovasc Disord. 2025 Dec 1;25:864. doi: 10.1186/s12872-025-05272-9 (PMC12699888; doi:10.1186/s12872-025-05272-9)
Supplement: Supplementary file 1 — Supplementary Material 1 [file 12872_2025_5272_MOESM1_ESM.docx]

**Supplementry Tables**

**Supp table (1): Correlation between miR-19b level and laboratory investigations among HF patients**

| **Studied variables** | **miR-19b** | |
| --- | --- | --- |
|  | **R** | **P value** |
| **Age (years)** | 0.043 | 0.781 |
| **Ejection fraction** | 0.821 | **<0.001*** |
| **Total cholesterol** | -0.062 | 0.685 |
| **HDL-C** | -0.081 | 0.596 |
| **LDL-C** | -0.054 | 0.727 |
| **Triglycerides** | 0.011 | 0.945 |
| **Hb** | 0.309 | **0.039*** |
| **WBCs** | 0.139 | 0.363 |
| **Platelets** | 0.152 | 0.320 |
| **ALT** | -0.204 | 0.179 |
| **AST** | -0.219 | 0.148 |
| **Urea** | -0.234 | 0.122 |
| **Creatinine** | -0.071 | 0.644 |
| **HbA1c** | 0.160 | 0.293 |
| **MiR-21** | -0.724 | **<0.001*** |
| **MiR-208a** | -0.789 | **<0.001*** |

**Supp table (2): Correlation between miR-21 level and laboratory investigations among HF patients**

| **Studied variables** | **miR-21** | |
| --- | --- | --- |
|  | **R** | **P value** |
| **Age (years)** | 0.090 | 0.558 |
| **Ejection fraction** | -0.793 | **<0.001*** |
| **Total cholesterol** | 0.311 | **0.038*** |
| **HDL-C** | -0.152 | 0.318 |
| **LDL-C** | 0.385 | **0.009*** |
| **Triglycerides** | -0.034 | 0.824 |
| **Hb** | -0.215 | 0.156 |
| **WBCs** | -0.189 | 0.214 |
| **Platelets** | -0.044 | 0.772 |
| **ALT** | 0.182 | 0.231 |
| **AST** | 0.187 | 0.219 |
| **Urea** | 0.311 | **0.038*** |
| **Creatinine** | 0.160 | 0.239 |
| **HbA1c** | -0.047 | 0.760 |
| **MiR-208a** | 0.764 | **<0.001*** |

**Supp table (3): Correlation between miR-208a level and laboratory investigations among heart failure patients**

| **Studied variables** | **miR-208a** | |
| --- | --- | --- |
|  | **R** | **P value** |
| **Age (years)** | 0.008 | 0.961 |
| **Ejection fraction** | -0.892 | **0.001*** |
| **Total cholesterol** | 0.238 | 0.115 |
| **HDL-C** | -0.109 | 0.477 |
| **LDL-C** | 0.284 | 0.058 |
| **Triglycerides** | -0.002 | 0.992 |
| **Hb** | -0.185 | 0.224 |
| **WBCs** | -0.184 | 0.228 |
| **Platelets** | -0.080 | 0.602 |
| **ALT** | 0.013 | 0.932 |
| **AST** | 0.066 | 0.664 |
| **Urea** | 0.167 | 0.273 |
| **Creatinine** | 0.184 | 0.226 |
| **HbA1c** | -0.138 | 0.366 |

**Supp table (4): Validity for NT-proBNP, miR-19b, miR-21 and miR-208a to discriminate HFpEF from HFmrEF**

| **Studied variables** | **AUC** | **P value** | **Cutoff point** | **Sensitivity (%)** | **Specificity (%)** | **PPV**  **(%)** | **NPV**  **(%)** |
| --- | --- | --- | --- | --- | --- | --- | --- |
| **NT-proBNP (pg/ml)** | 0.773 | 0.011^*^ | >729 | 86.67 | 80.0 | 81.2 | 85.7 |
| **miR-19b** | 0.964 | <0.001^*^ | ≤11.2 | 93.33 | 86.67 | 87.5 | 92.9 |
| **miR-21** | 0.701 | 0.045^*^ | >5.5 | 70.00 | 76.67 | 64.3 | 62.5 |
| **miR-208a** | 0.978 | <0.001^*^ | >10.3 | 93.33 | 93.33 | 93.3 | 93.3 |

**AUC:** Area under the curve

**PPV:** Positive predictive value

**NPV:** Negative predictive value

**Supp table (5): Validity for NT-proBNP, miR-19b, miR-21 and miR-208a to discriminate HFmrEF from HFrEF**

| **Studied variables** | **AUC** | **P value** | **Cutoff point** | **Sensitivity (%)** | **Specificity (%)** | **PPV**  **(%)** | **NPV**  **(%)** |
| --- | --- | --- | --- | --- | --- | --- | --- |
| **NT-proBNP (pg/ml)** | 0.831 | 0.002* | >842 | 80.0 | 73.33 | 75.0 | 78.6 |
| **miR-19b** | 0.998 | <0.001* | ≤3.8 | 100.00 | 93.33 | 93.7 | 100.0 |
| **miR-21** | 0.924 | <0.001* | >14.8 | 93.33 | 86.67 | 87.5 | 92.9 |
| **miR-208a** | 0.987 | <0.001* | >19 | 93.33 | 93.33 | 93.33 | 93.33 |

**AUC:** Area under the curve

**PPV:** Positive predictive value

**NPV:** Negative predictive value
